# The role of affective touch in mental illness: a systematic review of CT fiber dysregulation in psychological disorders and the therapeutic potential of CT fiber stimulation

**DOI:** 10.3389/fpsyt.2025.1498006

**Published:** 2025-03-25

**Authors:** Martina Papi, Davide Decandia, Daniela Laricchiuta, Debora Cutuli, Livia Buratta, Maurizio Peciccia, Claudia Mazzeschi

**Affiliations:** ^1^ Department of Philosophy, Social Sciences and Education, University of Perugia, Perugia, Italy; ^2^ Laboratory of Experimental and Behavioral Neurophysiology, Scientific Institutes for Research, Hospitalization, and Healthcare (IRCCS) Santa Lucia Foundation, Rome, Italy; ^3^ Department of Psychology, University Sapienza of Rome, Rome, Italy

**Keywords:** C tactile afferent, psychological disorder, psychological treatment, affiliative touch, social touch, affective touch therapies

## Abstract

**Introduction:**

Over the past few decades, research on affective touch has clarified its impact on key psychological functions essential for environmental adaptation, such as self-awareness, self-other differentiation, attachment, and stress response. These effects are primarily driven by the stimulation of C-tactile (CT) fibers. Despite significant advancements in understanding the fundamental mechanisms of affective touch, its clinical applications in mental health remain underdeveloped. This systematic review aims to rigorously assess the scientific literature on the relationship between CT fiber stimulation and psychological disorders, evaluating its potential as a therapeutic intervention.

**Methods:**

This systematic review was conducted in accordance with PRISMA guidelines. A search was performed in the EMBASE, PubMed, and Web of Science databases for articles published in the last 10 years. The review focused on two main aspects: (1) potential dysregulation of CT fibers in individuals with psychological disorders, and (2) psychological treatments based on CT fiber stimulation and their psychological and functional outcomes.

**Results:**

Most studies investigating CT fiber dysregulation in psychological disorders reported sensory alterations, with patients rating affective touch as less pleasant than healthy controls. These differences were often associated with dysregulation in the reward network and interoceptive processing, with several studies suggesting reduced insular cortex activation as a contributing factor. Regarding psychological treatments, only a limited number of studies analyzed therapies based on CT fiber stimulation. Despite methodological variations and differences in psychological diagnoses, the available evidence suggests that affective touch therapies can effectively reduce symptom severity and improve interoception across different psychological conditions.

**Discussion:**

The findings underscore the potential of affective touch as a therapeutic avenue for psychological disorders. However, given the dearth of studies on this topic, further analyses are necessary to fully understand its mechanisms and clinical efficacy. Expanding research in this area could provide valuable insights into functional impairments related to CT fiber dysregulation and support the development of targeted interventions for mental health treatment.

## 1 Introduction

Affiliative touch, characterized by slow and gentle gestures such as caresses and particularly effective when applied in a repetitive, rhythmic manner, constitutes a fundamental aspect of interpersonal sensorimotor interaction (1, 2). An expanding body of contemporary research on affective touch elucidates the underlying mechanisms of this specific form of skin contact, emphasizing its crucial role in a range of evolutionarily fundamental functions (1, 3). These functions include attachment, stress regulation, body representation, differentiation between self and others, and body ownership (2, 4–14).

The effects of affective touch are mediated by the activation of C-tactile (CT) unmyelinated fibers, a specialized class of low-threshold mechanoreceptors located in hairy skin that optimally respond to slow and gentle touch (15). CT fibers conduct signals at a velocity of approximately 1 m/s, significantly slower than myelinated Aβ fibers, which are responsible for processing discriminative touch (2). Experimental studies indicate that brush strokes applied at velocities between 1 and 10 cm/s, particularly at skin temperature, are consistently rated as more pleasant compared to those delivered at either slower or faster speeds (16). Microneurography studies have shown that the optimal velocity for activating CT fibers is between 1 and 10 cm/s, while velocities exceeding 10 cm/s activate only a limited number of CT fibers (17).

CT fiber activation is associated with the release of oxytocin (5) and dopamine (18), enhancing pleasure, alleviating pain, and reinforcing the attachment between caregiver and infant (5–7, 10). Research has demonstrated that skin-to-skin contact is correlated with elevated peripheral oxytocin levels in both parents and infants (19, 20) and that oxytocin levels peak after approximately 30 min of continuous, rhythmic stroking in both adults (2) and children (10). Notably, affective touch plays a crucial role in maintaining physiological stability in newborns, as evidenced by stable heart rate variability, suggesting its involvement in autonomic self-regulation and stress reduction (21). During stress-inducing situations, such as the Still Face paradigm, infants exhibit increased self-touch behaviors, highlighting the significance of affective touch in self-soothing mechanisms (22). Furthermore, infants engage in spontaneous self-touch, which contributes to the development of an early sense of self and body ownership (23, 24).

The CT fiber system establishes indirect connections with the posterior insular cortex (12, 25), primary (S1) and secondary (S2) somatosensory cortices, as well as the prefrontal cortex (11, 26) and precisely these connections may support the role of affective touch not only in the early development of interoceptive awareness but also in the integration of interoceptive and exteroceptive signals—a process fundamental to the emergence of bodily self-representation and the distinction between self and others (8, 13, 14, 27–29). In infants, gentle stroking has been found to activate both the S1 and the posterior insular cortex (30–32). Conversely, children raised in institutional care, who typically receive limited physical contact, often exhibit heightened sensitivity to sensory input, an increased prevalence of sensory processing difficulties, behavioral and psychological disorders, and, in some cases, an aversion to touch (33–36). These findings suggest that CT-mediated touch supports early sensorimotor and cognitive development as well as serves as a foundational element for relational and affective experiences throughout life.

On such a basis, a growing body of clinical research has explored the therapeutic potential of affective touch, demonstrating its efficacy in the treatment of various psychological disorders (37–41), in which the processes of body representation, differentiation between self and others, and body ownership are crucial. Despite this evidence, the implementation of affective touch in mental healthcare remains underdeveloped. A notable disparity exists between the substantial body of research on affective touch and the limited evidence-based clinical practices integrating this knowledge (42). Affective touch interventions are therapeutic techniques involving the application of gentle, non-intrusive physical contact, such as light stroking or holding, designed to promote emotional and psychological wellbeing. These interventions specifically engage CT afferents and include techniques such as therapeutic massage (43), Kangaroo Mother Care for preterm infants (44), affect-regulating massage therapy (ARMT) (45), psycho-regulatory massage therapy (PRMT) (46), mechanical affective touch therapy (MATT) (47, 48), and amniotic therapy (AT) (49). These therapies are utilized in various clinical settings, including pediatrics, geriatrics, psychiatry, and psychosomatic medicine, to enhance both mental and physical health.

The objective of this systematic review is to examine the scientific literature on the relationship between CT fiber stimulation and psychological disorders using a rigorous methodology. Specifically, we aim to (1) review studies assessing the potential dysregulation of CT fiber-mediated somatosensory processing in individuals with various psychiatric conditions, and (2) evaluate current psychological treatments that leverage CT fiber stimulation, analyzing their psychological and functional outcomes. By synthesizing existing findings, this review seeks to enhance the empirical framework for understanding the role of affective touch in psychopathology and its therapeutic potential in mental health interventions.

## 2 Materials and methods

### 2.1 Protocol

**Figure 1:**
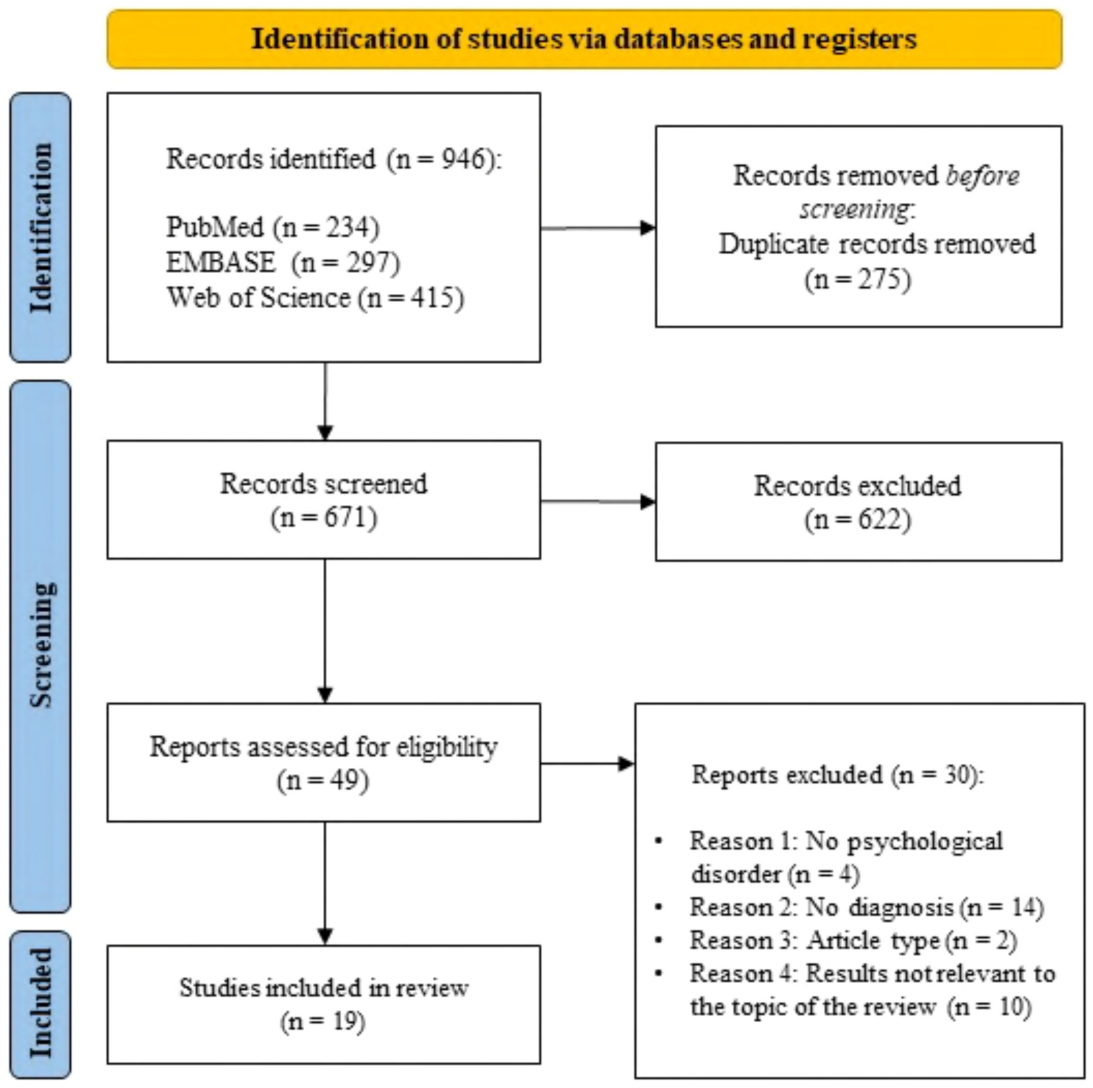
Search flow diagram according to PRISMA guidelines.

This systematic review was produced as stated by the Preferred Reporting Items for Systematic Reviews and Meta-Analyses (PRISMA) guidelines (50). The PRISMA flowchart of the study selection is described in **Figure 1**.

### 2.2 Search strategy and study selection

The systematic search of the literature was conducted in three databases—PubMed, EMBASE, and Web of Science—to screen articles published in the 10 years prior to the research date (27 February 2024), which focused on the following areas of interest: “psychological intervention” and “affective touch and CT fiber stimulation.” After keywords selection, the search was performed within “Title and abstract” in PubMed and EMBASE, and within “All fields” in Web of Science. In the following, advanced searches for each database are reported:

– PubMed advanced search: ((psychological therap* OR psychological treatment* OR psychological intervention* OR psychotherapy OR psychiatric) AND (affective touch OR affiliative touch OR C tactile fiber* OR C-tactile OR tactile C)).
– EMBASE advanced search: (((psychological AND therap* OR psychological) AND treatment* OR psychological) AND intervention* OR ‘psychotherapy’/exp OR psychotherapy OR psychiatric) AND (‘affective touch’/exp OR ‘affective touch’ OR (affective AND (‘touch’/exp OR touch)) OR ‘affiliative touch’ OR (affiliative AND (‘touch’/exp OR touch)) OR ((‘c’/exp OR c) AND tactile AND fiber*) OR ‘c tactile’ OR ‘tactile c’ OR (tactile AND (‘c’/exp OR c))).
– Web of Science advanced search: (((psychological therap* OR psychological treatment* OR psychological intervention* OR psychotherapy OR psychiatric) AND (affective touch OR affiliative touch OR C tactile fiber* OR C-tactile OR tactile C))).

The screening was independently performed by two different authors.

### 2.3 Inclusion and exclusion criteria

The PICOS model was used to determine the inclusion criteria:

– P (population): “individuals with a diagnosis of psychological disorder.”
– I (intervention): “CT fiber stimulation.”
– C (comparators): “individuals without a psychological diagnosis (i.e., control and placebo groups).”
– O (outcome): “psychological, neuromorphological, and biological differences in the effect of C-tactile fiber stimulation and the treatment effects of CT fiber stimulation.”
– S (study design): “observational studies, cohort studies, clinical trials, cross-sectional studies, and case–control studies.”

All human studies were included in this systematic review despite the study design. Conversely, all studies including animal models or *in vitro* and *in silico* studies were excluded. We excluded narrative reviews, systematic reviews, meta-analyses, and book chapters. In order to properly stick to the research question, we excluded articles that did not include patients whose diagnosis was stated and involved a validated method.

### 2.4 Risk of bias assessment

Two researchers (M.P. and D.D.) assessed the methodological quality of each included study using the Revised Cochrane Risk-of-Bias tool for randomized trials (RoB 2). This tool was designed to evaluate the risk of bias in randomized controlled trials (51). It consists of five key items, addressing five domains: selection bias, reporting bias, performance bias, attrition bias, and other potential sources of bias.

### 2.5 Data extraction

After defining the inclusion and exclusion criteria and having completed the selection of studies, data were extracted and summarized in **Tables 1**–**5**, reporting the following information:

– Description of all selected studies including sample size, gender, ethnicity, age, psychological diagnosis, diagnosis tool, comorbidity, medical treatment, and exclusion criteria (**Table 1**).
– Methodologies (**Table 2**) and results (**Table 3**) of studies evaluating CT fiber stimulation to assess potential CT fiber dysregulation in individuals with a diagnosis of psychological disorder. The tables include information regarding stimulation protocol, psychological assessment, neuroimaging analyses, electrophysiologic activity recordings, and other analyses (e.g., subjective evaluation of the pleasantness of tactile stimulation through Likert scale or a visual analog rating scale).
– Methodologies (**Table 4**) and results (**Table 5**) of studies in which CT fiber stimulation was used as psychological treatment. The tables include information regarding stimulation protocol, psychological assessment, neuroimaging analyses, electrophysiologic activity recordings, and biological analyses (e.g., heartbeat tracking).

**Table 1:** Summary of the sample description of all studies.

Article	Sample Size	Gender	Age	Psychological Diagnosis	Diagnosis Tool	Comorbidity	Medical Treatment	Exclusion Criteria
Arnold et al., 2020 (45)	57 outpatients with depression. Intervention group: 30; Control group: 27	Control group: 22 F; 5 M. Intervention group: 22 F; 8 M	Intervention group: 45.2 ± 9.43 years, range: 24–60. Control group: 44.9 ± 12.29 years, range: 19–64	Diagnosis of a mild to moderate depressive episode, including the following diagnoses: depressive episode, recurrent depressive disorder	International Classification of Diseases - 10 (ICD-10)	Somatic symptoms	During the study, almost half of the participants, in both groups, were under psychotherapeutic treatment	Subjects with an acute comorbid medical condition, eczematous skin disease, patients with marked varicose veins or venous thrombosis, pregnant women, and subjects who were simultaneous participating in another clinical trial
Baumgart et al., 2020 (46)	66 patients with chronic unspecific back pain or somatoform disorders. Intervention group: 33; Control group: 33	No information(NI)	Age range: 18–75 years	Diagnosis of chronic unspecific back pain or somatoform disorders	ICD-10	Depression	NI	Subjects with open wounds, inflammatory disorders, ongoing application for pension, limited ability for consent to study participation
Carpenter et al., 2022 (47)	22 outpatients with anxiety disorders	16 F, 5 M, 1 non-binary or trans	Mean age: 37.3 ± 14.8 years, range: 18–59	Diagnosis of a moderate to severe level of current anxiety disorder, including the following diagnoses: generalized anxiety disorder, major depressive disorder episode, panic disorder, social anxiety disorder (generalized or non-generalized), obsessive–compulsive disorder, post-traumatic stress disorder	Diagnostic and Statistical Manual of Mental Disorders - 5 (DSM-5), Mini International Neuropsychiatric Interview (MINI)	Major depression	16 subjects were on stable doses of antidepressants/anxiolytics, 6 subjects were free of psychiatric medications	Patients with bipolar I and primary psychotic disorders, contraindications to magnetic resonance imaging (MRI), significant neurological conditions, hospitalization for a psychiatric disorder within the past 6 moths, change in psychotropic medication within the past month, dermatological condition
Cascio et al., 2016 (52)	33 children with autism spectrum disorder; 20 children with other development disabilities; 56 children with typical development	NI	NI	Group with autism: autism spectrum disorder. Group with development disabilities: a known genetic syndrome associated with intellectual disability, a nonspecific developmental delay or developmental delay associated with prematurity	Autism Diagnostic Interview – Revised (ADI-R), Autism Diagnostic Observation Schedule (ADOS), DSM-IV	NI	NI	Subjects with a diagnosis of any genetic condition associated with autism, seizure disorder, mental age below 6 months, receipt of psychopharmacological treatments that might alter sensory responses
Croy et al., 2018	70 subjects presenting to outpatients’ psychotherapy; 69 controls	Intervention group: 60 F, 10 M. Control group: 56 F, 13 M	Intervention group: 46.0 ± 12.0 years, age range: 24–70. Control group: 45.6 ± 12.5 years, range: 21–67	Mood and affective disorders, somatoform disorders, disorders of personality, post-traumatic stress disorder, anxiety disorders	ICD-10	NI	Antidepressant: 30 patients, 0 controls; tranquilizers: 9 patients, 0 controls	Subjects with diabetes, disease of liver, kidney, neurological disease, history of brain surgery, and subjects with skin disease on tested area
Crucianelli et al., 2016 (54)	25 patients with anorexia nervosa; 30 controls	All female participants	Anorexia nervosa patients: 24 ± 12.75 years. Control group: 26 ± 7.25 years	Anorexia nervosa	DSM-IV, Structured Clinical Interview for DSM-5 Disorders (SCID)	NI	16 patients self-reported being on medication	Subjects with any skin condition, any substance abuse and left-handed subjects. Regarding Intervention group: patients with a history of any other axis I clinical disorder, a body mass index out of the normal range, and any indications on psychometric assessment of clinical depression or anxiety disorder
Crucianelli et al., 2020 (14)	27 participants with restrictive type of anorexia nervosa; 24 patients remitted from restrictive type of anorexia nervosa; 27 controls	NI	Anorexia nervosa patients: 28.40 ± 10.44; subjects remitted from anorexia nervosa: 24.25 ± 4.51; control group: 24.48 ± 4.61	Anorexia nervosa	DSM-5	Group with anorexia nervosa: depression, bipolar disorder, anxiety disorder, obsessive–compulsive disorder, borderline personality disorder; group remitted from anorexia nervosa: depression, anxiety disorder, borderline personality disorder	Selective serotonin reuptake inhibitors, tranquillizers in subjects with anorexia nervosa and subjects remitted from anorexia nervosa	Subjects with any skin condition, any substance abuse and left-handed individuals
Davidovic et al., 2018 (56)	25 patients with anorexia nervosa; 25 controls	All female participants	Anorexia nervosa patients: 20.3 ± 2.2 years. Control group: 21.2 ± 2.1 years	19 patients diagnosed of restrictive type of anorexia nervosa; 4 patients diagnosed with a binge-eating/purging type of anorexia nervosa	DSM-IV (SCID)	Depression	13 patients used psychoactive medication: fluoxetine, sertraline, olanzapine, quetiapine, venlafaxine, propiomazin, lamotrigine, and lisdexamfetamine	NI
Frost-Karlsson et al., 2022 (57)	25 participants with anorexia nervosa; 29 subjects with autism spectrum disorder; 57 controls	Anorexia nervosa patients: 25 F. Autism spectrum disorder patients: 18 F, 11 M	Anorexia nervosa patients: 21.3 ± 2.6 years; Control group of anorexia nervosa: 22.5 ± 2.3 years. Autism spectrum disorder patients: 24.1 ± 5.3 years; Control group of autism spectrum disorder: 24.1 ± 3.5 years	Anorexia nervosa; autism spectrum disorder	DSM-5	NI	Participants were either free of psychotropic medications or on stable medication with antidepressants	Patients with anorexia nervosa: schizophrenia or psychotic disorder, autism spectrum disorder diagnosis, bipolar disorder, alcohol/drug use disorder, ongoing treatment with antipsychotic, severe head injury, birth before 33 weeks of gestation, hearing impairment, earlier epilepsy or seizure, claustrophobia, pregnancy, cognitive disabilities. Patients with autism spectrum disorder: psychiatric conditions, chronic pain
Germani et al., 2019 (49)	Single patient with schizophrenia	Male participant	31 years old	Schizophrenia	DSM-IV (SCID-I)	NI	Intervention plan did not include medicines	NI
Gonsalves et al., 2022 (48)	20 outpatients with anxiety disorder	14 F, 6 M	Mean age: 35.80 ± 14.72 years, range: 18–65	Anxiety disorder	MINI	Depression	Patients were medication-free or on a stable regimen of psychotropic medications	Subjects who were psychiatrically hospitalized, attempted suicide (within the previous 6 months), had MRI contraindications, were diagnosed with neurological conditions
Lee Masson et al., 2020 (58)	21 adults with autism spectrum disorder; 21 neurotypical adults	All male participants	Autism spectrum disorder patients: 25.0 ± 4.4 years. Control group: 23.9 ± 2.8 years	Autism spectrum disorder	DSM-IV, DSM-5	NI	Patients did not take psychotropic medications	Patients with autism spectrum disorder: comorbid neurological, psychiatric, genetic conditions. Control group: history of neurological, psychiatric, medical conditions known to affect brain structure and function
Löffler et al., 2022 (59)	25 patients with borderline personality disorder; 25 controls	All female participants	Borderline personality disorder patients: 31.28 ± 7.57 years; control group: 26.72 ± 8.57 years	Borderline personality disorder	DSM-IV (SCID), International Personality Disorder Examination (IPDE)	Borderline personality disorder patients: major depressive disorder (7), post-traumatic stress disorder (8), anorexia nervosa (0), other eating disorder (7). Other current mental disorder (20), more than one current mental disorder (11)	None of the subjects had on on-demand medication; regular psychotropic and pain medication were discontinued for at least 2 weeks (except for selective serotonin reuptake inhibitors)	Subjects with scars on the back of the left hand, diagnosis of bipolar I disorder, schizophrenia, insufficient speech comprehension, mental retardation, body mass index < 16.5, substance use disorder (within the last year), fibromyalgia, serious physical illness, severe brain disorder, and pregnancy
Maier et al., 2020 (60)	33 participants with low childhood maltreatment; 30 participants with medium childhood maltreatment; 29 participants with high childhood maltreatment	Low childhood maltreatment: 24 F, 9 M; Medium childhood maltreatment: 16 F, 14 M; High childhood maltreatment: 24 F, 5 M	Low childhood maltreatment: 25.7 ± 0.97 years; Medium childhood maltreatment: 29.53 ± 1.97 years; High childhood maltreatment: 28.35 ± 1.56 years. Total: 27.8 ± 8.50	Post-traumatic stress disorder	DSM-IV, The Clinician-Administered PTSD Scale (CAPS)	Depression	Subjects were medication-free	Subjects with psychotic disorders, neurological abnormalities, history of head trauma, use of psychotropic medications, and MRI contraindications
Mielacher et al., 2024 (61)	53 patients with major depressive disorder; 41 healthy controls	Major depressive disorder patients: 27 F, 26 M; Control group: 22 F, 19 M	Major depressive disorder patients: 41.58 ± 13.09 years. Control group: 40.61 ± 13.22 years	Unipolar major depressive disorder	DSM-IV, MINI	Anxiety	47 major depressive disorder patients received pharmacotherapy for the duration of the study: 18 selective serotonin reuptake inhibitors, 15 selective serotonin-norepinephrine reuptake inhibitor, 32 atypical antidepressant, 10 atypical antipsychotics, 11 anticonvulsants, 5 tricyclic antidepressants, 4 levothyroxine, 2 antihistamines, 1 benzodiazepine, 1 lithium, 1 monoamine oxidase inhibitor, 1 norepinephrine reuptake inhibitor	For all participants: suicidal ideation, psychotic symptoms, bipolar depression, substance abuse, eating disorders, post-traumatic stress disorder, personality disorders, neurological disorders, and MRI contraindications. Additional for controls: lifetime axis I or II psychiatric disorders, past or current psychopharmacological medication
Perini et al., 2021 (62)	26 patients with autism spectrum disorder; 25 typically developing control subjects	Autism spectrum disorder patients: 4 F, 22 M; Control group: 3 F, 22 M	Autism spectrum disorder patients, male subjects: 17.0 ± 1.1 years, range: 16–20; female subjects: 16.3 ± 0.5 years, range: 16–17. Control group, male subjects: 17.5 ± 1.7 years, range: 16–22; female subjects: 17.0 ± 1.0 years, range: 16–18	Autism spectrum disorder	DSM-IV or DSM-5	13 patients with autism spectrum disorder were diagnosed with attention-deficit/hyperactivity disorder, 3 with depression	6 participants assumed psychotropic medications	Subjects with neurological disorders, intellectual disability, past or current psychotic symptoms, insufficient knowledge of Swedish, functional magnetic resonance imaging (fMRI) contraindications, previous severe head injury, seizures, other significant medical illness, and premature birth. Additional for controls: no DSM axis I or II disorder in the past year and not taking any psychotropic medications
Schienle et al., 2024 (63)	70 patients with skin-picking disorder; 62 healthy controls	All female participants	Skin-picking disorder patients: 25.57 ± 6.28 years. Control group: 23.87 ± 5.59 years	Skin-picking disorder	DSM-5	47% of skin-picking disorder patients showed comorbid mental disorders, including anxiety disorders, depression, obsessive–compulsive disorder, and eating disorders	Three patients with skin-picking disorder took psychotropic medications: 2 selective serotonin reuptake inhibitors and 1 serotonin-norepinephrine reuptake inhibitors	Patients with skin-picking disorder: diagnoses of major depression with severe symptoms, substance abuse/dependence, borderline personality disorder, psychosis, and dermatological conditions. Control group: diagnoses of mental disorder, dermatological conditions, and psychotropic medications
Strauss et al., 2019 (64)	Experiment 1: 13 patients with post-traumatic stress disorder; 13 healthy controls. Experiment 2: 20 patients with post-traumatic stress disorder; 20 healthy controls	Experiment 1: all female participants. Experiment 2: 19 F, 1 M in both control subjects and post-traumatic stress disorder patients	Experiment 1, post-traumatic stress disorder patients: 41.9 ± 15.5 years, range: 20–55; Control group: 39.8 ± 7.8 years, range: 30–55. Experiment 2, post-traumatic stress disorder patients: 44.3 ± 10.7 years, range: 24–58; Control group: 40.3 ± 14.0 years, range: 24–58	Post-traumatic stress disorder	DSM-IV (SCID)	NI	NI	Experiment 1, control group: mental disorders through Patient Health Questionnaire (PHQ). Experiment 2, control group: current psychiatric/psychotherapeutic treatment, subjective suffering from mental disorder through Beck’s Depression Inventory (BDI-II) and PHQ, and history of childhood maltreatment through Childhood Trauma Questionnaire (CTQ)
Zoltowski et al., 2023 (65)	13 adults with autism spectrum disorder; 14 non-autistic participants	Autism spectrum disorder patients: 1 F, 12 M; Control group: 1 F, 13 M	Autism spectrum disorder patients: 28 years, range: 17–47. Control group: 30 years, range: 20–51	Autism spectrum disorder	ADOS	NI	NI	Subjects with history of medical conditions associated with autism, MRI contraindications

White portions identify studies related to CT fiber dysregulation in psychological suffering. Grayscale portions identify studies related to therapeutic intervention based on CT fiber stimulations.

**Table 2:** Summary of the methodological approach of studies on CT fiber functionality to assess potential CT fiber dysregulation in individuals with a diagnosis of psychological disorder.

Materials and Methods					
Article	Stimulation Protocol	Psychological Assessment	Neuroimaging	Electrophysiologic Activity Recording	Other
Cascio et al., 2016 (52)	Passive touch through three textures (unpleasant, pleasant, and social) at three different body sites: perioral face, dorsal forearm, and thenar palm. Experimenter administered all combinations twice (18 trials)	Cognitive evaluation: Stanford-Binet Intelligence Scales (SB5), Mullen Scales of Early Learning (MSEL). Sensory evaluation: Sensory Experiences Questionnaire (SEQ)	Not applicable (NA)	NA	Subjective evaluation of the pleasantness of tactile stimulation through a 5-point Likert scale. The examiner evaluated the immediate reaction after each trial to evaluate defensiveness
Croy et al., 2016 (53)	Passive touch in the left dorsal forearm by an examiner through a soft brush; 15 stroking stimuli were presented with different velocities (0.3, 1, 3, 10, and 30 cm/s)	Emotional evaluation: BDI-II, CTQ. Cognitive evaluation: Autism Spectrum Quotient (AQ). Sensory evaluation: unstandardized question concerning relationship status and frequency of contact	NA	NA	Subjective evaluation of the pleasantness of tactile stimulation through a visual analog rating scale (VAS). Self-report evaluation of the pleasantness of five pictures with different hedonic quality
Crucianelli et al., 2016 (54)	Two contemporary stimulations: visual and tactile stimulation. Visual stimuli: 45 photos of women with different expressions (smiling, rejecting, neutral). Tactile stimuli: passive touch in the left forearm through a soft brush. In total, 45 trails in two velocities: 3 cm/s and 18 cm/s	Emotional evaluation: Depression Anxiety Stress Scale (DASS) - 21	NA	NA	Subjective evaluation of the pleasantness of tactile stimulation using a rating scale after each trial
Crucianelli et al., 2020 (14)	Passive touch in the left forearm through a soft brush. 25 trials in total with 3 trials for each stimulation speed: two CT-optimal (3 and 6 cm/s), one borderline (9 cm/s), and two not CT-optimal (18 and 27 cm/s)	Sensory evaluation: Eating Disorder Inventory-2 (EDI-2), Body Awareness Questionnaire (BAQ). Emotional evaluation: Toronto Alexithymia Scale (TAS-20)	NA	NA	Before sensory trail, participants rated the pleasantness of a hypothetical touch with different material. Subjective evaluation of the pleasantness of tactile stimulation using a rating scale after each trial
Davidovic et al., 2018 (56)	Custom-built robotic linear tactile stimulation: passive touch in the right dorsal forearm through a brush, with a velocity of 2 cm/s. Skin stroking was alternated with skin indentation	Emotional evaluation: BDI	Tactile stimulation was executed during structural and functional MRI (resting state)	NA	Subjective evaluation of the pleasantness of tactile stimulation after each session
Frost-Karlsson et al., 2022 (57)	Three tactile conditions (10 times for each condition): other-touch (slow stroke in the left arm from the examiner), self-touch, and object-touch (participants stroked a pillow)	Psychological evaluation: AQ, Social Touch Questionnaire (STQ)	Tactile stimulation was executed during structural and functional MRI	NA	NA
Lee Masson et al., 2020 (58)	Pleasant (brush) and unpleasant (elastic band) touch stimulation (5 cm/s) in the forearm (rest trial and four blocks with eight trials for each block)	Psychological evaluation: STQ, Social Responsiveness Scale for Adults (SRS-A) (in previous study)	Tactile stimulation was executed during structural and functional MRI. ROIs: primary somatosensory cortex (S1), parietal operculum (PO), insula, superior temporal gyrus (STG), amygdala, anterior cingulate cortex, and orbitofrontal cortex	NA	Subjective evaluation of the pleasantness of tactile stimulation through a Likert-like scale
Löffler et al., 2022 (59)	Pleasant touch stimulation (3 cm/s) on the back of the hand through a brush and a custom apparatus	Symptomatologic evaluation: Borderline Symptom List (BSL-23). Emotional evaluation: BDI, State-Trait-Anxiety Inventory (STAI). Dissociation evaluation (before and after touch stimulation): Dissociation-Tension Scale acute (DSS-4)	NA	Electromyography (EMG) to evaluate the acoustic startle response; startle probe occurred nine times with and nine times without tactile stimulation	Subjective evaluation of the perceived intensity and valence of tactile stimulation through a VAS and touch perception task (TPT). Quantitative sensory testing (QST) protocol to assess touch sensitivity at baseline. Thermal sensory analyzer device to assess warm perception and heat pain
Maier et al., 2020 (60)	Manual administration of passive touch in subjects’ shins (slow: 5 cm/s; fast: 20 cm/s)	Psychological evaluation: CTQ and STQ. Emotional evaluation: BDI-II, Perceived Stress Scale (PSS)	Tactile stimulation was executed during fMRI	NA	Interpersonal distance paradigm: four consecutive trails to assess the ideal/unconformable interpersonal distance. Social Touch Paradigm: subjective evaluation of the pleasantness of tactile stimulation after each trial
Mielacher et al., 2024 (61)	Manual administration of passive touch in participants’ shins (slow: 5 cm/s; fast: 20 cm/s; 20 trials for each condition) with cotton gloved hands by an examiner	Psychological evaluation: CTQ and STQ. Emotional evaluation: STAI. Symptomatologic evaluation: Hamilton Depression Rating Scale (HDRS-17) (weekly), BDI-II (before-after the treatments, every 4 weeks over a 12-week follow-up period)	Tactile stimulation was executed during functional and structural MRI (after admission, 24 days later)	NA	Subjective evaluation of the pleasantness of tactile stimulation after each trial
Perini et al., 2021 (62)	Three tasks: social processing task, affective picture processing task, and tactile stimulation task. Tactile stimulation task: tactile strokes (slow: 3 cm/s; fast: 30 cm/s; 15 trials for each condition) through a brush in the dorsal part of the left forearm	Psychological evaluation: AQ and STQ, Five Health-Relevant Personality Traits (HP5) Inventory, questionnaires about participants’ family, illness, and medications. Emotional evaluation: BDI-II, Beck Anxiety Inventory (BAI). Sensory evaluation: Social Responsiveness Scale-2 (SRS-2), Edinburgh Handedness Inventory (EHI)	Tactile stimulation task was executed during fMRI (resting-state)	NA	Subjective evaluation of the pleasantness and the intensity of tactile stimulation (slow/fast) through a VAS; affective touch awareness
Schienle et al., 2024 (63)	Passive touch with closed eyes through a soft brush (slow: 3 cm/s; fast: 30 cm/s)	Symptomatologic evaluation: Skin-Picking Scale revised (SPS-R), The Milwaukee Inventory for the Dimensions of Adult Skin Picking (MIDAS), Skin Picking Impact Scale (SPIS)	Tactile stimulation task was executed during structural and functional MRI	NA	Sensorial evaluation: two-point discrimination test. Subjective evaluation of the pleasantness and the arousal of tactile stimulation after each condition
Strauss et al., 2019 (64)	Experiment 1: passive touch in the left dorsal forearm, presented in five conditions (three trials for each condition): impersonal touch (brush) at 30 cm/s (fast), impersonal touch at 3 cm/s (slow), interpersonal (palm of the experimenter) at 3 cm/s, interpersonal visually shielded at 3 cm/s, self-touch. Experiment 2: passive touch in the left dorsal forearm in four runs with four conditions (impersonal and interpersonal touch, slow and fast touch)	Experiment 1, psychological evaluation for all participants: CTQ and PTSD Checklist (PCL), the Questionnaire of dissociative symptoms (FDS-20), Positive and Negative Affect Schedule (PANAS); psychological evaluation for control group: PHQ. Experiment 2, psychological evaluation for all participants: CTQ, PANAS, and BDI-II; psychological evaluation for control group: PHQ; psychological evaluation for post-traumatic stress disorder (PTSD) patients: PCL and FDS-20	Tactile stimulation was executed during fMRI	NA	Experiment 1: subjective evaluation of the pleasantness and the intensity of tactile stimulation through a VAS. Participants reported any memories or intrusions occurring during the stroking at the end of the condition. Experiment 2: subjective evaluation of the pleasantness and the intensity of tactile stimulation through a 10-point scale
Zoltowski et al., 2023 (65)	Passive touch in the right forearm (5 cm/s); stimulation occurred with three texture type: pleasant (soft brush), neutral (burlap), unpleasant (mesh); two runs including two block per texture type	Cognitive evaluation: Wechsler Abbreviated Scales of Intelligence (WASI). Sensory evaluation: Adult Sensory Profile	Tactile stimulation was executed during structural and functional MRI	NA	Subjective evaluation of the pleasantness of tactile stimulation

**Table 3:** Summary of the results of studies on CT fiber functionality to assess potential CT fiber dysregulation in individuals with a diagnosis of psychological disorder.

Results				
Article	Psychological Assessment	Neuroimaging	Electrophysiologic Activity Recordings	Other
Cascio et al., 2016 (52)	Subscales ADI-R for the current behavior: negative correlation between the self-report evaluation of the pleasant material and nonverbal communication impairment and a trend for a positive correlation between rating for the unpleasant material and social impairment in patients with autism spectrum disorder (ASD). Positive correlations between defensiveness at the forearm/perioral sites and current social impairment in ASD group	NA	NA	Lower evaluations of pleasant and social materials in patients with ASD and other development disabilities (DD); unpleasant material’s evaluation did not differ between groups. Significant main effect of group and bodily site in the defensiveness scores given by the examiner; higher defensiveness scores in ASD and DD groups compared to subjects with typical development (TD). Significantly higher defensive’s reaction in the ASD group compared to the TD group at perioral and forearm. Negative correlations between defensiveness and pleasantness
Croy et al., 2016 (53)	Negative association in both groups between affective touch awareness and AQ; positive association in both groups between affective touch awareness and history of childhood maltreatment. Diagnosis of personality disorder was associated in both group with the reduction of overall touch pleasantness. Lower interpersonal contact reported from patients compared to controls	NA	NA	Lower pleasant ratings in all velocities in patients compared to controls. Higher ratings of middle velocities in both groups compared to other velocities
Crucianelli et al., 2016 (54)	Significantly higher depression, anxiety, and stress in patients with anorexia nervosa (AN) compared to controls. No correlation between DASS-21 scores and pleasantness scores or between DASS-21 scores and the difference between slow and fast touch in each group	NA	NA	Significantly lower evaluation of pleasantness of CT-optimal touch in AN patients compared to controls. Significantly higher evaluation of pleasantness of slow touch compared to fast touch. Significantly higher evaluation of pleasantness of passive touch when accompanied with accepting faces compared to neutral or rejecting faces
Crucianelli et al., 2020 (14)	TAS-20: significantly higher alexithymia ratings in AN patients compared to subjects remitted from anorexia nervosa (RAN) and controls. EDI-2: significantly higher interoceptive awareness’ score in the AN group compared to RANs and controls. No correlations were found between EDI-2 scores and the pleasantness of touch in the various speeds. BAQ was a predictor of pleasantness for the CT-optimal touch in AN and RAN group, not in the control group	NA	NA	Imagined touch: significant difference in evaluation of pleasantness of smooth/harsh materials between AN group and controls. Sensory trails: significantly higher evaluation of pleasantness of slow and borderline touch compared to fast touch. Significantly lower evaluation of pleasantness of touch in AN and RAN patients compared to controls
Davidovic et al., 2018 (56)	No correlation between the evaluation of pleasantness of touch and BDI scores in AN patients	Skin stroking condition: increased activity in S1, secondary somatosensory cortex (S2), bilateral insula in both groups; no significant group differences were found. Increased activity in bilateral lateral occipital cortex in controls, decreased activity in AN patients. Skin stroking–skin indentation condition: increased activity in left caudate nucleus, bilateral frontal pole, bilateral precuneus, and right temporal pole in controls, and decreased activity in AN patients	NA	Significantly lower evaluation of pleasantness in AN patients compared to controls
Frost-Karlsson et al., 2022 (57)	Highest AQ and STQ scores in the ASD group, intermediate values in AN, and lowest values in controls	Significantly higher activation in the AN group as compared to the ASD group and controls. Self-touch condition: increased response in the claustrum, cingulate cortex, frontal and temporal areas, S1, striatum, insula, parahippocampal gyrus in AN patients compared to controls; higher activity in parahippocampal gyrus, superior and middle temporal gyrus, middle frontal gyrus, cingulate, putamen, and insula in AN patients compared to ASD patients. Other-touch condition: increased activity in S1 and STG in AN subjects compared to ASD patients and controls	NA	NA
Lee Masson et al., 2020 (58)	Negative association between STQ and SRS-A. Functional connectivity between the semantic and the limbic network: marginally associated with higher SRS-A score in pleasant condition; significant association with lower positive attitude into social touch in unpleasant condition	ASD group: fewer stimulus-dependent changes in functional connectivity based on pleasant/unpleasant touch as compared to controls; significant difference in the functional connectivity strength between left and right PO; hypoconnectivity between PO and insula, hyperconnectivity between the semantic and limbic networks	NA	NA
Löffler et al., 2022 (59)	No significant difference in the variation of general dissociation (DSS-4) in patients with borderline personality disorder (BPD) with and without PTSD	NA	Affect-modulated acoustic startle response did not differ significantly between groups in perceived valence and intensity of touch	Significantly lower perceived valence and intensity of touch in patients with BPD as compared to controls; negative correlation between symptoms severity and the evaluation of the pleasantness of touch. Dissociative states in BPD: negative correlation between state dissociation prior to the session and the perceived intensity of touch; significant increase in state dissociation from pre/post touch; positive correlation between the perceived valence of touch and changes in ownership of the stimulated arm. Qualitative aspects of TST: rougher and firmer perception of touch in BPD group compared to controls
Maier et al., 2020 (60)	Higher social aversion (STQ) associated with lower comfort evaluations of slow and fast touch. Social touch aversion mediated the effect of childhood maltreatment (CM) on the perceived comfort of slow and fast touch; indirect effect of social aversion on the association between CM and stress scores	Low-CM subjects: slow touch produced widespread activations including hippocampus and insula. High-CM subjects: increased cortical responsiveness to fast touch in right S1 and right posterior insula; decreased limbic reactivity to slow touch in the right hippocampus compared to low-CM subjects. High-CM subjects compared to low-CM subjects: significant reductions of gray matter in the left–right hippocampus, the left–right S1, the left–right posterior insula, and the left amygdala	NA	Interpersonal distance paradigm: high-CM participants preferred a larger interpersonal distance compared to low-CM ones; no significant difference between medium and high CM. Preference for larger interpersonal distance was associated with lower comfort evaluations of fast touch. Negative association between the reactivity of right S1 and right posterior insula and the perceived comfort of fast touch. Social touch paradigm: high CM participants judged fast touch as less comforting compared to medium and low CM subjects. Main effects of announcement of touch (present/absent), touch velocity, a touch velocity-by-CM group interaction
Mielacher et al., 2024 (61)	HDRS-17: significant reduction of symptom severity in major depressive disorder (MDD) patients over time; 23 patients were classified as responders. STQ: higher social touch aversion in MDD group compared to controls	Reduced neural reactivity to interpersonal touch, regardless of velocity or time (pre–post) of touch, in the bilateral nucleus accumbens and bilateral caudate nucleus in the MDD group compared to controls. Association between speed, time and group in the left putamen. Treatment response: reduced reactivity during social touch in the right caudate nucleus in non-responders; reduced activity in the left anterior insula in non-responders during slow touch compared to responders	NA	Social touch comfort ratings: main effects of speed (low-fast), group; patients with MDD judged social touch as less comfortable compared to controls, not after slow touch
Perini et al., 2021 (62)	No association was found between affective touch awareness and AQ scores in ASD patients and controls	Fast touch caused an increased S1 activation and slow touch caused an increased postcentral gyrus activation. Positive correlation between the difference in brain activity for slow/fast touch in the right posterior superior temporal sulcus and affective touch awareness in the control group compared to patients	NA	No significant difference in the evaluation of pleasantness or intensity between groups; slow touch was judged as more pleasant and less intense; significant main effect of speed for pleasantness perceived and for intensity ratings. Affective touch awareness: significantly lower affective touch awareness scores in ASD compared to controls
Schienle et al., 2024 (63)	Higher rating on SPS-R, SPIS, and MIDAS in skin-picking disorder (SPD) patients compared to controls	In SPD patients, compared to controls: increased activity in right supramarginal gyrus (SMG) and the right angular gyrus (ANG) during slow touch; higher connectivity between the SMG and the right medial ANG; negative correlation between valence evaluation and the reactivity of right insula; negative correlation between the urge to skin-picking and the reactivity of the left SMG. Control group showed deactivation of the inferior and medial frontal gyrus during slow touch	NA	Significant effect of group for pleasure, arousal, and the urge to perform skin-picking: SPD patients showed less pleasure, greater arousal, and a higher urge. Main effect of velocity of touch in pleasure, arousal, and urge: slow touch determined more pleasure, less arousal, and less urge compared to fast touch. Discriminative touch score did not differ between groups
Strauss et al., 2019 (64)	Experiment 1: higher scores of childhood maltreatment (CTQ), PTSD symptomatology (PCL), dissociation severity (FDS), more negative and fewer positive emotions in patients with PTSD compared to controls. Experiment 2: higher scores of childhood traumatization (CTQ), more negative and fewer positive emotions (PANAS) in PTSD patients compared to controls; patients showed high symptomatology, dissociation severity, depression (PCL, FDS, and BDI-II)	Experiment 2: Increased reactivity of STG in all conditions, especially in interpersonal touch, in PTSD patients compared to controls; significant association between activity of STG and pleasantness evaluation. Higher activation of hippocampus and the visual cortex for impersonal compared to interpersonal touch in the PTSD group. Interpersonal condition in patients: negative correlation between the PCL subscale negative alterations and the reduced hippocampal activation; positive correlation between PCL subscale arousal and the reduced hippocampal activation	NA	Experiment 1: main effect of group with a significant interaction between group and touch type; lower evaluations of pleasantness of the two interpersonal conditions in the PTSD group; 5 patients and 1 control reported negative memories after touch (3 patients’ memories were related to trauma). Experiment 2: significantly lower evaluation of pleasantness in both touch conditions, especially in interpersonal touch, in PTSD patients compared to controls; higher evaluation of intensity of touch in PTSD patients compared to controls; significant interaction velocity and group: patients did not differentiate between slow and fast touch regarding pleasantness or intensity of touch
Zoltowski et al., 2023 (65)	Adult sensory profile: significant association between higher “sensory seeking” scores and lower posterior insula blood oxygenation level-dependent (BOLD) responses in the early phase of the pleasant condition; high correlation between early- and late-phase responses and sensory profile domains	Unpleasant stimulation: higher BOLD response in the ASD group compared to controls in the early phase of stimulation in PO, left postcentral gyrus, and middle gyrus; late-phase activations in frontal gyrus for the ASD group and in opercular/insular regions for controls. Neutral stimulation: greater BOLD responses in the early-intermediate phases in controls, unlike the ASD group, especially in a para-cingulate cluster, S1, PO, and inferior frontal gyrus; significant BOLD responses in the late phase in both groups: frontal pole in controls and paracingulate in ASD. Pleasant stimulation: no significant BOLD responses in the early/intermediate phase, unlike controls (S1, PO); significant BOLD response in middle temporal gyrus in the ASD group; quite a decrease in S1 compared to controls	NA	No significant relationship was found between BOLD response and texture pleasantness evaluation for any region, texture, and response phase; the strongest association was found between the posterior insula responses in the intermediate phase and comfort evaluation for all three textures

**Table 4:** Summary of the methodological approach of studies using CT fiber stimulation as a psychological treatment.

Materials and Methods					
Article	Treatment Protocol	Psychological Assessment	Neuroimaging	Electrophysiologic Activity Recordings	Biological Analyses
Arnold et al., 2020 (45)	Intervention group: one affect-regulating massage therapy (ARMT) session (60 min) weekly over 4 weeks, with preheated oil to 35°C. Control group: one progressive muscle relaxation (PMR) session weekly over 4 weeks	Emotional evaluation: Hamilton Depression Scale (HAMD), Bech-Rafaelsen-Melancholia-Scale (BRMS), VAS, semi-structured interview; data were collected at baseline and at the end of the therapy	NA	NA	NA
Baumgart et al., 2020 (46)	Intervention group: two psycho-regulatory massage therapy (PRMT) session weekly (10 treatments). Pressure: soft to moderate; Speed: decreasing to a speed of 10^−3^ cm per second; Direction: connected body areas ending with cranial to caudate strokes; Rhythm: constant contact with patients. Control group: two classical massage therapy (CMT) session weekly (10 treatments). Pressure: soft to strong; Speed: slow speed; Direction: from the origin to the beginning of muscle; Rhythm: not defined	Emotional evaluation: BDI-II. Pain assessment: Hamburg Pain adjective list (HSAL), Oswestry Disability Index (ODI). Data were collected at baseline, on the 5th and 10th treatment, and at follow up (3 months)	NA	NA	NA
Carpenter et al., 2022 (47)	Two sessions of auto-administrated mechanical affective touch therapy (MATT) per day for 4 weeks (20 min per session). Device for home use: mechanical stimulation behind each ear via piezoelectric disks mounted on a headset; power was generated from an MP3 player that converted signal to vibrations	Emotional evaluation: Generalized Anxiety Disorder-7 (GAD-7), BDI, PSS, and DASS. Multidimensional Assessment of Interoceptive Awareness (MAIA). Other evaluation: Systematic Assessment for Treatment Emergent Events (SAFTEE). Self-reported scales to assess symptomatology at baseline, week 2, and at the end of the treatment	fMRI data were collected at baseline and at the end of the treatment	Resting electroencephalogram (EEG): data were collected before and after a baseline MATT session; before the last MATT session	NA
Germani et al., 2019 (49)	Four amniotic therapy (AT) sessions weekly, conducted for 3 years; setting: warm water	Emotional evaluation: Positive and Negative Syndrome Scale (PANSS), Global Assessment of Functioning Scale (GAF). Functioning was assessed at baseline, after 1, 2, and 3 years of intervention	NA	NA	Interoceptive accuracy was measured trough heartbeat tracking task
Gonsalves et al., 2022 (48)	Two sessions of auto-administrated mechanical affective touch therapy (MATT) per day for 4 weeks. Device for home use: mechanical stimulation behind each ear via piezoelectric disks mounted on a headset; power was generated from an MP3 player that converted signal to vibrations	Emotional evaluation: GAD-7, PSS, BDI, DASS, and MAIA. Data were collected at baseline, week 2, and week 4	Resting-state fMRI at baseline (15 min, before and after the first MATT session) and at the end of the treatment	EEG data collected at baseline	NA

**Table 5:** Summary of the results of studies using CT fiber stimulation as a psychological treatment.

Results				
Article	Psychological Assessment	Neuroimaging	Electrophysiologic Activity Recordings	Biological Analyses
Arnold et al., 2020 (45)	VAS: significant increase in treatment effects in the intervention group compared to controls, especially in stress/tension, hopelessness, internal unrest, pain sensation, psychomotor retardation, and unpleasant physical sensation. HAMD: stronger symptomatologic reduction in the intervention group compared to controls; items with moderate effect size: depressive mood, somatic symptoms. Items that achieved a statistically significant difference in BRMS: emotional retardation and sleep disorders	NA	NA	NA
Baumgart et al., 2020 (46)	Reduction of depression intensity and pain in the intervention group compared to controls, especially at follow-up. Decrease in depression severity in the intervention group from moderate to minimal on average	NA	NA	NA
Carpenter et al., 2022 (47)	MAIA: significant correlation between the degree of increase in acute frontal alpha power (FAP) at baseline and the degree of mindfulness increase after 4 weeks in attention and self-regulation. Significant correlation between the level of occipital theta power (OTP) increases and symptom’s reduction in depression, stress, and anxiety	NA	Alpha power: higher FAP was correlated with greater symptoms on the DASS-Anxiety and DASS-Stress scales at baseline. After chronic MATT, greater reductions in perceived stress were correlated with dampening of FAP. Acute stimulation caused a significant increase in FAP, and the extent of change was associated with mindfulness (MAIA) and enhanced attention regulation at week 4. Theta Power: at baseline, frontal theta power correlated with perceived stress	NA
Germani et al., 2019 (49)	Significant decrease in positive symptom scores, negative symptom scores; increase in global functioning	NA	NA	Increase in interoceptive accuracy at the end of the treatment compared to the baseline
Gonsalves et al., 2022 (48)	MATT was associated with a significant decrease in mean scores in GAD-7, PSS, BDI, and DASS, and a significant increase in MAIA global score. Increase in positive functional connectivity between the cingulate cortex and the left anterior supramarginal cortex was associated with decrease in DASS (depression and stress)	Higher positive connectivity between resting-state functional connectivity and default mode network regions predicted stronger clinical improvement at the end of the treatment. Increase in right anterior insula functional connectivity to salience and interoceptive regions after the first MATT session. Positive correlation between right insula and left precentral time courses after stimulation. Negative correlation between anterior cingulate-to-anterior supramarginal functional connectivity and cortical thickness in the right insula and the left cingulate	NA	NA

## 3 Results

### 3.1 Selected studies investigating the role of affective touch in psychological suffering

The bibliographic research offered a total of 946 articles that met the search criteria (**Figure 1**). PubMed search produced 234 articles, EMBASE search produced 297 results, and 415 articles were provided by Web of Science. After excluding 275 duplicate records, 671 papers were screened by reading the title and abstract; out of these, 622 articles were excluded and 49 publications were admitted for the full-text screening. After the second screening, 30 reports were discarded due to the inconsistency with our inclusion criteria. The remaining 19 articles were included in the present systematic review. Out of these, 14 studies primarily focus on an exploratory analysis of neurobiological and morphological aspects associated to CT fiber functionality in different psychopathological conditions (52–65). Furthermore, 5 studies focus on CT fiber stimulation as part of psychological treatment (45–49). The description of 19 studies is reported in **Table 1**.

### 3.2 The dysregulation of CT fibers in psychological suffering

The methods and results of analyses on CT fiber functionality to assess potential CT fiber dysregulation in patients suffering from various psychological disorders are summarized in **Tables 2**, **3**.

#### 3.2.1 Autism spectrum disorder

Based on a comprehensive literature screening, five studies have investigated the potential dysregulation of CT fibers in individuals diagnosed with autism spectrum disorder (ASD) (52, 57, 58, 62, 65). These studies examined neuroimaging evidence (57, 58, 62, 65) and subjective perceptions of pleasantness (52, 58, 62, 65) in response to standardized tactile stimulation, employing various experimental protocols. Specifically, the studies differed in the tactile texture of stimulation and the velocity of touch. Researchers explored pleasant and unpleasant touch (52, 58, 65), as well as neutral touch using burlap fabric (65). Additionally, self-touch and object-touch (57), along with social-touch interactions (52, 57), were assessed.

Perini et al. (2021) (62) administered CT stimulations at velocities of 3 and 30 cm/s, while Zoltowski et al. (2023) (65) and Lee Masson et al. (2020) (58) employed a 5 cm/s velocity. In all studies, affective touch was applied to the participants’ forearm (57, 58, 62, 65), except for Cascio et al. (2016) (52), who stimulated three bodily sites characterized by varying levels of CT fiber innervation: the perioral face, dorsal forearm, and thenar palm.

Furthermore, the phenomenon of sensory defensiveness—characterized by heightened emotional reactions and hyperresponsiveness—was analyzed, revealing significantly greater defensive reactions in children with ASD and other developmental disabilities compared to control groups. In the ASD cohort, defensiveness reactions were particularly elevated in the perioral and forearm regions, which are densely innervated by CT fibers. Notably, defensiveness was negatively correlated with perceived pleasantness.

Lee Masson et al. (2020) (58) identified a negative correlation between the connectivity of the semantic network (i.e., lateral temporal lobe) and the limbic network (i.e., bilateral hippocampus and amygdala), as assessed via functional magnetic resonance imaging (fMRI), and attitudes toward social touch. These attitudes were evaluated using the Social Touch Questionnaire (STQ) and the Social Responsiveness Scale for Adults (SRS-A) (66). Specifically, the STQ assesses individuals’ perceptions of touch-related social interactions in daily life, particularly regarding the quality of caressing experiences. In neurotypical control subjects, stronger functional connectivity was observed between the parietal operculum (PO) and the right insula during pleasant touch. In contrast, individuals with ASD exhibited reduced modulation in these regions, characterized by hypo-connectivity between the PO and insula and hyper-connectivity between the semantic and limbic networks.

Perini et al. (2021) (62) examined subjective evaluations of pleasantness and perceived intensity in response to tactile stimulation, using these measures as proxies for affective touch awareness and its neural correlates. The analysis found no significant differences in comfort or intensity ratings between ASD and control participants. Across all groups, slow touch was rated as more pleasant and less intense than fast touch. However, only in neurotypical individuals did affective touch awareness positively correlate with neural responses in the right posterior superior temporal sulcus.

Zoltowski et al. (2023) (65) utilized fMRI to investigate blood oxygenation level-dependent (BOLD) responses during tactile stimulation. In individuals with ASD, a heightened BOLD response was observed in the early phase of stimulation within the PO, left postcentral gyrus, and middle frontal gyrus during unpleasant touch. In contrast, neurotypical individuals exhibited a graded response pattern. Regarding neutral stimulation, participants with ASD showed no significant BOLD responses in the aforementioned areas until the late stimulation phase, whereas neurotypical subjects exhibited early-phase activation. At this later phase, participants with ASD demonstrated significant BOLD responses in the paracingulate cortex, while controls exhibited activity in the frontal pole. In response to pleasant touch, individuals with ASD displayed significant BOLD responses only in the late phase, with increased activation in the middle temporal gyrus and decreased activity in the S1 relative to controls. Furthermore, an inverse relationship was observed between early-phase responses to pleasant stimulation and sensory-seeking behaviors in the ASD group, as assessed using the Adult Sensory Profile (67).

Finally, one study (57) utilized fMRI to investigate self–other distinction during tactile stimulation in individuals with ASD, those with anorexia nervosa (AN), and neurotypical controls. In the ASD group, no regions exhibited increased BOLD activity compared to the AN and control groups, suggesting a potential alteration in the neural mechanisms underlying self–other differentiation in ASD.

#### 3.2.2 Anorexia nervosa

The analysis of potential CT fiber dysregulation in patients diagnosed with AN identified four relevant studies (54–57). In all studies, patients with AN and healthy controls received CT fiber stimulation and subsequently rated the perceived pleasantness of the touch, except in Frost-Karlsson et al. (2022) (57). Additionally, in two studies, tactile stimulation was administered during fMRI scanning (56, 57).

In Frost-Karlsson et al. (2022) (57), neuroimaging data revealed that during self-touch, individuals with AN exhibited significantly higher activation in the claustrum, cingulate cortex, frontal and temporal regions, S1, striatum, insula, and parahippocampal gyrus compared to controls. Moreover, greater activation was observed in the parahippocampal gyrus, superior and middle temporal gyri, middle frontal gyrus, cingulate cortex, insula, and putamen in patients with AN compared to those with ASD. During other-touch conditions, subjects with AN showed significantly higher activity in S1 and the superior temporal gyrus compared to both patients with ASD and controls.

Davidovic et al. (2018) (56) also utilized fMRI to examine brain responses during CT fiber stimulation, which was administered via a linear tactile stimulator. Participants received several trials of passive touch on the right forearm at a velocity of 2 cm/s, interspersed with blocks of static skin indentation. Self-reported ratings of comfort during skin stroking revealed a significantly lower evaluation of pleasantness in patients with AN compared to controls, with no correlation to depression scores. Whole-brain analysis showed a significantly reduced response to skin stroking versus skin indentation in patients with AN compared to controls, specifically in the left caudate nucleus, bilateral frontal pole, bilateral precuneus, and right temporal pole. Additionally, patients with AN exhibited significantly decreased activation in the bilateral lateral occipital cortex in response to skin stroking, whereas controls demonstrated increased activity in this region during tactile stimulation.

Crucianelli et al. (2016) (54) investigated the interplay between visual and tactile stimulation. Participants underwent CT fiber stimulation on the forearm via brush strokes at two different velocities, while simultaneously viewing images of individuals displaying critical/rejecting expressions, neutral expressions, or smiling faces. The study aimed to determine whether the perceived pleasantness of touch was modulated by the emotional quality of the observed facial expression. Patients with AN, who exhibited significantly higher levels of depression, anxiety, and stress compared to controls, reported significantly lower comfort ratings in response to CT-optimal stimulation. In both groups, exposure to smiling faces enhanced the perceived pleasantness of touch compared to neutral or rejecting facial expressions.

Similarly, another study (55) reported significantly lower pleasantness ratings for touch in patients with AN compared to controls. This study investigated tactile anhedonia in individuals with AN, those recovered from AN, and healthy controls. Participants completed the Interoceptive Awareness subscale of the Eating Disorder Inventory-2 and the Body Awareness Questionnaire (BAQ) before undergoing CT fiber stimulation on the forearm at three different velocities: CT-optimal (3 cm/s), non-optimal (30 cm/s), and borderline (9 cm/s), classified as such given that CT-optimal stimulation typically falls within the 1–10 cm/s range (16). Patients with AN exhibited significantly higher interoceptive awareness compared to controls. Importantly, body awareness was identified as a predictor of perceived comfort in response to CT-optimal touch within the clinical group.

#### 3.2.3 Post-traumatic stress disorder

The literature review identified three studies examining CT fiber stimulation in patients diagnosed with post-traumatic stress disorder (PTSD) (53, 60, 64). In these studies, researchers administered passive touch to individuals with PTSD and healthy controls under different conditions, varying both the velocity and method of stimulation. Croy et al. (2016) (53) applied stimulation at 0.3, 1, 3, 10, and 30 cm/s, while Maier et al. (2020) (60) utilized slow (5 cm/s) and fast (20 cm/s) velocities. Strauss et al. (2019) (64) conducted two experiments, exposing participants to combinations of impersonal and interpersonal touch, CT-optimal (3 cm/s) or non-optimal (30 cm/s) velocities, and self-touch. In two studies (60, 64), tactile stimulation was administered during fMRI scanning. All studies required participants to rate the perceived pleasantness of CT fiber stimulation. Additionally, Maier et al. (2020) (60) included an interpersonal distance paradigm to assess participants’ preferred interpersonal distance, while Strauss et al. (2019) (64) (Experiment 1) invited participants to report any memories evoked during touch stimulation.

In Maier et al. (2020) (60), the study sample comprised adults with varying levels (low, moderate, and high) of childhood maltreatment (CM). Participants were screened for lifetime psychiatric disorders using the DSM-IV (Diagnostic and Statistical Manual of Mental Disorders, Fourth Edition) and for current PTSD using the Clinician-Administered PTSD Scale. Individuals with high CM exhibited a preference for greater interpersonal distance and rated fast touch as significantly less pleasant compared to those with no or moderate CM. They also reported heightened discomfort during tactile stimulation, particularly in response to fast touch. This discomfort was associated with increased activation in the right S1 and posterior insula in response to fast touch, as well as reduced neural responses to slow touch in the right hippocampus. Additionally, significant reductions in gray matter volume were observed in the bilateral hippocampus, bilateral S1, bilateral posterior insula, and the left amygdala in participants with high CM.

Similarly, Strauss et al. (2019) (64) conducted two experiments with distinct participant groups, utilizing various psychological assessment tools. Across both experiments, the clinical groups exhibited significantly higher levels of PTSD symptomatology, CM, and dissociative symptoms compared to healthy controls. Moreover, the clinical groups reported experiencing fewer positive and more negative emotions than controls. In both experiments, all touch conditions were rated as less comfortable by the clinical group, with interpersonal touch receiving the most negative evaluation. In Experiment 1, one control participant and five individuals with PTSD reported intrusive memories during touch, three of which were trauma-related. In addition to the assessments conducted in Experiment 1, the second experiment found that PTSD patients exhibited high levels of depressive symptoms and reported perceiving touch as more intense. Furthermore, they did not differentiate between slow and fast touch in terms of pleasantness. In individuals with PTSD, touch aversion was associated with reduced hippocampal responses and increased activation in the superior temporal gyrus. Moreover, hippocampal response was negatively correlated with symptoms of negative affect and positively correlated with arousal symptoms.

Finally, Croy et al. (2016) (53) investigated CT fiber stimulation in patients recruited from an outpatient psychotherapy clinic and healthy controls. The clinical group comprised individuals diagnosed with various psychopathological conditions, including PTSD. Following CT fiber stimulation, patients rated touch as significantly less pleasant than controls. Consistent with previous findings, mid-range velocities, particularly CT-optimal stimulation (3 cm/s), were perceived as the most pleasant. The study further revealed that higher CM scores and lower autism spectrum quotient scores were associated with greater affective touch awareness. Although not statistically significant, diagnoses of PTSD and personality disorders contributed to this model.

#### 3.2.4 Personality disorders

Two studies investigated CT fiber stimulation in patients diagnosed with personality disorders (PD) (53, 59). In the study conducted by Croy et al. (2016) (53), the authors reported a reduction in the overall perceived pleasantness of touch in the clinical group compared to healthy controls. Similarly, in the second study (59), participants received tactile stimulation at a CT-optimal velocity (3 cm/s) and were asked to rate the perceived pleasantness of the touch. This study included patients with borderline personality disorder (BPD) and healthy controls.

Participants underwent psychological assessments and electromyographic recording to measure the acoustic startle response, which served as a physiological correlate of affective response. Patients with BPD reported significantly lower ratings of touch valence and intensity, describing the stimulation as rougher and firmer compared to controls. Moreover, in patients with BPD, symptom severity was inversely correlated with the perceived intensity of touch. A significant increase in dissociative state from pre- to post-tactile stimulation was also observed. Additionally, in the BPD group, the perceived valence of touch was positively correlated with changes in the sense of ownership of the stimulated arm.

#### 3.2.5 Major depressive disorder

Two studies investigated CT fiber stimulation in patients diagnosed with major depressive disorder (MDD) (53, 61). In Croy et al. (2016) (53), as previously discussed, the clinical group included aggregated data from individuals with various psychopathological conditions, without distinguishing specific diagnoses. However, the global analysis found no significant association between depression severity and affective touch awareness.

In contrast, the second study (61) specifically examined patients with MDD and healthy controls. Participants received manually administered passive touch at slow (5 cm/s) and fast (20 cm/s) velocities during fMRI sessions, conducted both at hospital admission and 24 days later. They were asked to rate the perceived comfort of the tactile stimulation. Most patients received pharmacotherapy throughout the study and periodically completed depression inventories to assess clinical improvement. Patients with MDD exhibited greater aversion to social touch, lower comfort ratings for tactile stimulation, and reduced neural responses to interpersonal touch in the nucleus accumbens, caudate nucleus, and putamen compared to healthy controls. Antidepressant treatment led to a reduction in clinical symptoms over time. However, patients who did not respond to therapy demonstrated persistently reduced activity in the caudate nucleus, anterior insula, and putamen.

#### 3.2.6 Skin-picking disorder

The literature review identified only one study analyzing CT fiber stimulation in patients with skin-picking disorder (SPD) (63). The authors investigated tactile processing in individuals with SPD and healthy controls, who received passive touch at CT-optimal (3 cm/s) and non-optimal CT (30 cm/s) velocities during fMRI scanning. Patients with SPD scored higher on all scales compared to controls, reporting lower comfort, greater arousal, and a stronger urge to engage in skin-picking, with a significant main effect of touch velocity. Neuroimaging analyses revealed increased activity in the right supramarginal gyrus (SMG) and angular gyrus (ANG) in response to CT-optimal velocity touch. Additionally, greater connectivity was observed between the SMG and medial frontal gyrus, as well as between the ANG and SMG. A negative correlation emerged between insular activity and the positive valence of touch in patients with SPD compared to controls. Lastly, whereas healthy controls exhibited deactivation of the inferior and medial frontal gyri during CT-optimal velocity touch, this pattern was not observed in the SPD group.

### 3.3 Therapeutic potential of CT fiber stimulation in psychological suffering

The methodologies and results of investigations into therapeutic intervention based on CT fiber stimulation and affective touch in patients with psychopathological disorders are summarized in **Tables 4**, **5**.

#### 3.3.1 Risk of bias assessment

**Figure 2:**
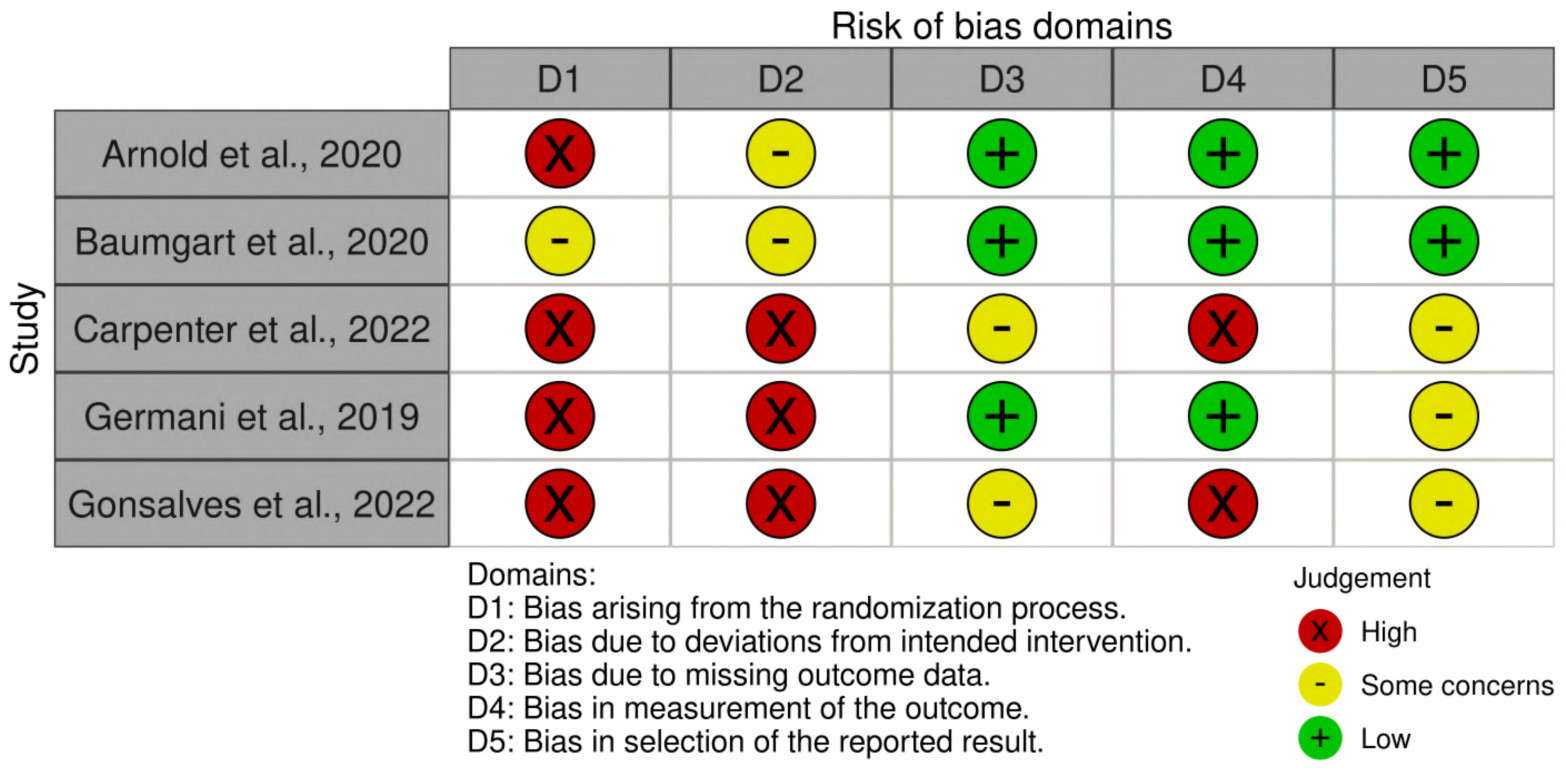
Risk of bias summary. Green represents low risk; red represents high risk; yellow represents some concerns.

Risks of bias were judged based on the Cochrane guidance, as shown in **Figure 2**.

A risk of bias assessment was conducted on studies proposing an intervention model based on affective touch, resulting in five articles (45–49). Two studies (45, 46) were rated as low risk of bias in domains 3, 4, and 5, leading to an overall low or moderate risk of bias. In contrast, three studies (47–49) were rated as high risk of bias in most domains, particularly Gonsalves et al. (48) and Carpenter et al. (47). These articles (47, 48) presented significant limitations, as they relied on the same sample without a control group, which prevented the randomization and double-blind control. Lastly, Germani et al. (49) conducted a single-case study, which is not fully applicable to the proposed quality appraisal. As a result, it showed some criticism, particularly in domains 1 and 2. Overall, the risk of bias assessment highlighted methodological strengths in some studies, while others exhibited significant limitations, underscoring the need for more rigorous research to strengthen the evidence on affective touch interventions.

#### 3.3.2 Depressive disorder

Arnold et al. (2020) (45) used the ARMT, which was conducted in a quiet room using preheated massage oil (35°C), and involved a sequence of ventral, diagonal, and symmetrical gentle strokes administered in both supine and prone positions. The treatment consisted of one ARMT session (60 min) per week over four consecutive weeks. Regarding ARMT (45), the authors observed a greater reduction in depressive symptomatology in the intervention group compared to controls, as measured by the Hamilton Depression Scale (HAMD) at the end of the treatment, especially in depressive mood and somatic symptoms. The visual analogue scale (VAS) for self-assessment showed significantly greater treatment effects in the intervention group compared to controls, with larger pre–post differences in stress/tension, hopelessness, internal unrest, pain sensation, psychomotor retardation, and unpleasant physical sensation. The Bech–Rafaelsen Melancholia Scale (BRMS) revealed significant differences in emotional retardation and sleep disorders at the end of the treatment; in particular, the ARMT group showed greater symptom reduction compared to the control group.

#### 3.3.3 Somatoform disorder

Baumgart and colleagues (2020) (46) used the PRMT, which involved continuous whole-body massage, patient positioning (supine and prone), and the use of preheated oil. PRMT began with three partial massages before progressing to a full-body massage with different intensities of touch, ranging from soft to moderate pressure. The protocol included a full-body massage, ending with strokes from cranial to caudal areas while decreasing the velocity to 10^−3^ cm per second. Participants underwent two PRMT sessions (30 to 60 min each) per week over five consecutive weeks. In this study (46), the authors examined the effect of PRMT on somatoform and depressive symptoms using BDI-II. Pre–post assessment scores revealed a significant and sustained reduction in depression severity in the intervention group compared to controls, decreasing from a moderate to minimal level on average. Additionally, after completing PRMT, patients with somatoform disorder experienced sustained pain reduction compared to controls (46).

#### 3.3.4 Anxiety disorder

The two studies focusing on anxiety disorders (47, 48) employed a home-based device that enabled participants to self-administer MATT, which delivers soft vibrations to the bilateral mastoid processes. The prototype included round ceramic piezoelectric actuators that converted the signal into mechanical stimulation behind the patients’ ears. These actuators were attached to a metal headset and powered by an MP3 signal generator. Carpenter et al. (2022) (47) chose an isochronic 10-Hz wave as a stimulation pattern, cycling 2 s on and 2 s off. Gonsalves et al. (2022) (48) tailored the stimulation intensity to participants’ preferences, selecting the level immediately above the threshold of perception. In both studies, participants were instructed to perform two self-administrated MATT sessions per day (20 min each) for four consecutive weeks. In both studies (47, 48), a significant decrease in mean symptom scores (47) was observed, along with an increase in Multidimensional Assessment of Interoceptive Awareness (MAIA) total scores in patients (47, 48). After MATT, scores on the General Anxiety Disorder-7 (GAD-7), Perceived Stress Scale (PSS), DASS, and BDI significantly decreased (48). Additionally, reductions in depression and stress DASS subscale scores were correlated with increased positive functional connectivity between the cingulate cortex and the left anterior supramarginal cortex after the completion of MATT (48). EEG data revealed that higher frontal alpha power (FAP) was associated with greater stress and anxiety DASS subscale scores at baseline (47). At the end of the treatment, greater reductions in perceived stress were correlated with dampening of FAP. Additionally, increased occipital theta power (OTP) was significantly associated with a reduction in depression, stress, and anxiety scores. Also, the extent of the increase in acute FAP at baseline correlated with the degree of mindfulness improvement at the end of the treatment in MAIA subscales, specifically in attention regulation and self-regulation (47). fMRI evidence revealed an increase in right anterior insula functional connectivity to salience and interoceptive regions following the first MATT session (48). At the end of the treatment, stronger functional connectivity between pain-processing regions (anterior insula, thalamus, and mid-cingulate area), anxiety regions (amygdala), and the default mode network was predictive of greater clinical improvement (48).

#### 3.3.5 Schizophrenia

Germani and colleagues (2019) (49) proposed an AT, to be performed in warm water. Each patient was supported by a therapist, enabling the creation of an amniotic holding: a continuous fluctuation between skin-to-skin contacts and separation movements (49). The protocol provided four weekly AT sessions (90 min each), conducted over a period of 3 years. To assess the effect of AT in schizophrenia, researchers administered the Positive and Negative Syndrome Scale (PANSS) and the Global Assessment of Functioning Scale (GAF) (49). The authors reported a significant reduction in positive symptom scores, a decrease in negative symptomatology, and an improvement in global functioning. Furthermore, patients demonstrated higher levels of interoceptive accuracy at the end of the treatment compared to baseline.

## 4 Discussion

### 4.1 CT fiber dysregulation across psychological disorders: impact on touch perception, emotional processing, and clinical implications

We analyzed the potential CT fiber dysregulation, through psychological and functional evaluation, in patients with a diagnosis of psychological disorder (ASD, AN, PTSD, PD, MDD, and SPD). Taken together, many studies suggest an alteration of CT fiber sensory processing, resulting in lower perceived pleasantness rating of touch in subjects with psychopathological conditions (52–56, 59–61, 63, 64), while only one study (62) did not detect any difference in perceived comfort of touch between patients with ASD and healthy controls. Patients with ASD showed high defensiveness reaction (52), and subjects with MDD reported higher aversion to interpersonal touch (53, 61). Patients affected by PTSD revealed a higher estimation of the intensity of stroke (53, 60, 64), while patients with BPD were characterized by a lower evaluation of the intensity of touch, whose perception was rougher and firmer (53, 59). Subjects with SPD perceived the stimulation as less pleasant, more arousing, and evoking higher urge (63); lastly, a study underlined lower perceived comfort and tactile anhedonia as a persisting trait in individuals with AN and RAN, even during the recovery (54, 55). The heterogeneity in responses—ranging from heightened defensiveness in ASD and MDD to altered intensity perception in PTSD and BPD—raises intriguing questions about the underlying neurobiological mechanisms. One possibility is that these differences reflect disorder-specific alterations in somatosensory–affective integration. Moreover, the persistence of tactile anhedonia in AN and RAN, even during recovery, suggests that impairments in affective touch processing may not merely be symptomatic of acute pathology but could represent a stable trait-like feature, influencing long-term emotional regulation. This opens avenues for exploring whether interventions targeting affective touch—such as sensory-based therapies—could mitigate affective dysregulation in these conditions. Future research should investigate whether these alterations in CT fiber function contribute to broader patterns of social cognition and attachment, potentially influencing treatment responsiveness and prognosis. Patients affected by AN reported significantly lower activation in LOC, a hub of processing images of human bodies and self-representation (68). This alteration may reflect a disturbed body perception network. Moreover, a significant decrease in the left caudate nucleus’ activity in subjects with AN was found as compared to controls (56). Several selected studies corroborated the hypoactivation of the reward circuit and its components, involving patients diagnosed with AN (56), MDD (61), and PTSD (60, 64). According to Nestler and Carlezon (2006) (69), another study (61) investigated the association between reward network, affective touch, and MDD. The authors, through fMRI data, evidenced a decreased neural activation in the reward system in patients compared to controls, specifically in nucleus accumbens, caudate nucleus, and putamen, independently of the velocity of stimulation (61). Moreover, in responder patients, the hypoactivation of the nucleus accumbens and caudate nucleus persisted after the end of the antidepressant treatment. Those non-responder patients showed decreased activity in the caudate nucleus, putamen, and anterior insula during social touch both before and after the treatment. The alteration in neural processing of the reward network, combined with social aversion, can promote social isolation (61). The shared involvement of the reward circuit across AN, MDD, and PTSD highlights a potential transdiagnostic mechanism linking affective dysregulation and maladaptive social functioning. Future research should explore whether targeting reward system dysfunction could enhance treatment outcomes. Understanding the interplay between reward processing and affective touch may offer novel therapeutic insights, particularly for individuals resistant to conventional treatments. As regards patients with PTSD, Maier et al. (2020) (60) reported a sensory cortical (S1, posterior insula) hyperreactivity to discriminative touch (i.e., fast touch) and a limbic (hippocampus) hypoactivation to affective touch (i.e., slow touch) during fMRI. Hyperreactivity of the posterior insula may indicate increased salience detection, while hippocampal hypoactivation may impair affective touch encoding due to reward-associated cells (70). In another study (64), the authors suggested that the decreased hippocampal response may be associated with a coping mechanism based on voluntary suppression of unwanted memories (71). This deactivation was also connected with increased activity in the STG, as well as touch aversion (64). Moreover, a significant reduction in gray matter volume in the hippocampus, S1, insula, and amygdala in patients with PTSD was revealed (60). The amygdala is rated as a core region in processing CT fiber stimulation (26), and it is involved in social behavior, valence and salience of stimuli, and reward processing (72). In patients affected by ASD, the authors (58) revealed dysfunctional cortical communication in several regions implicated in the processing of affective touch, including alteration in STG, bilateral PO, and insula (73), via fMRI scanning. The authors suggest that these findings can be imputed to an insufficiency of stimulus-dependent modulation in regional connectivity patterns during the skin strokes (58). Patients with ASD exhibited hyper-connectivity between semantic and limbic networks, linked to social touch aversion, and hypo-connectivity between PO and the insula (58). Asaridou et al. (2024) (74) demonstrated that autistic individuals generally have heightened tactile sensitivity but found no autism-specific sex differences, implying that certain sensory traits might serve as universal autism markers. Osório et al. (2021) (75) revealed that autistic female patients exhibit more severe sensory processing difficulties, particularly in auditory and balance-related domains, which could aid in refining diagnostic criteria for female autism. These findings underscore the importance of considering sex differences in sensory and affective processing, as they have direct implications for both clinical practice and psychological research. In fact, Schirmer et al. (2022) (76) found that while men and women exhibit similar sensory pleasantness to touch, women display higher interpersonal comfort with unfamiliar touch and more negative affective associations, suggesting that touch may serve as a more relevant coping mechanism for them. The results suggest that diagnostic and therapeutic approaches should be tailored to account for sex-specific sensory profiles, particularly in autism, where female presentation is often overlooked. In SPD, greater insula activity correlated with lower affective touch ratings (63). Moreover, an increased SMG–ANG connectivity in patients was found, while controls showed deactivation in the inferior and middle frontal gyrus in comparison to patients with SPD. With SMG and ANG being two areas involved in attentional control, the authors hypothesized that the self-stimulation in SPD can be useful to redirect attentional resources from external stressors to inner sensations (63). Leknes and Tracey (2008) (77) evidenced a neurobiological similarity in affective somatosensory processing of pain and pleasure. This affinity is determined by the common involvement of areas such as the insula, amygdala, prefrontal cortex, and orbitofrontal cortex, as well as the common modulation of the opioid and the dopamine system. According to these findings, another study (59) suggested that both processes can be altered in BPD, in terms of lower sensitivity, according to a cortico-limbic (i.e., top-down modulation) dysregulation pathway (78). Löffler et al. (2022) (59) found a significant increase in state dissociation from pre- to post-stimulation in subjects with BPD, and the perceived valence of touch was related to the change in ownership of the stimulated arm. This association was not seen in the non-stimulated arm. The authors suggest that a decrease in body ownership experiences could be associated with an unpleasant perception of touch (59).

### 4.2 Neurobiological effects and clinical benefits of CT fiber stimulation in treating psychological disorders: from stress reduction to interoception enhancement

The previously mentioned findings contribute to the evaluation of CT fiber stimulation protocols (MATT, AT, ARMT, and PRMT) and their therapeutic potential in psychological suffering. Taken together, each protocol produced a decrease in symptom severity at the end of the treatment (45–49). In particular, two studies (47, 48) investigated MATT’s therapeutic effects via resting-state fMRI and EEG. MATT exploited insula activity and the associated interoceptive training through CT fiber stimulation. Through resting-state fMRI, Gonsalves et al. (2022) (48) evidenced that greater functional connectivity between the insula and the amygdala and the default mode network at baseline was related to a stronger decrease in stress and anxiety symptoms at the end of the treatment. Acutely (after a single MATT session), the authors observed higher insula connectivity to salience and interoceptive regions. However, interoceptive awareness, measured at the end of the treatment (MAIA), did not change significantly, suggesting that it may require a longer time window than the MATT treatment (48). Chronic effects of MATT (i.e., after 4 weeks of treatment) were detected, via fMRI data, in greater connectivity between the mid-cingulate cortex and the lateral subnetwork of the default mode network, resulting in a decrease in stress and anxiety scores (DASS) (48). In addition, a previous study revealed an association between the interoceptive awareness, promoted by mindfulness techniques, and an increase in FAP (79). Based on this evidence, another study (47) revealed a significant association between FAP and the symptoms’ severity at baseline, while after MATT completion, the decrease in FAP was related to the greatest symptoms’ reduction, suggesting a ceiling effect. In line with the previous study, Germani et al. (2019) (49) focused on the key role of the posterior insula in interoception (80) as well as in identification–separation processes (i.e., self–other distinction) (49). The authors evinced a progressive implementation in global functioning and a decline in positive and negative symptoms during a 3-year-long AT. Moreover, the interoceptive accuracy, measured through heartbeat tracking task, was enhanced at the end of the treatment, suggesting a potential tool for reducing self-disorder in patients who have been diagnosed with schizophrenia (49). Similarly, applying the ARMT protocol, Arnold et al. (2020) (45) observed a significant decrease in depressive symptomatology in subjects with MDD as compared to controls, with larger differences in internal unrest, unpleasant physical sensation, pain sensation, and stress. The authors identified the therapeutic potential of ARMT in insula activation, able to normalize a disturbed interoception (81). A previous study (18) linked gentle touch to oxytocin release and lower cortisol, supporting reduced stress scores (45). Interestingly, a previous study (82) observed an increase in oxytocin levels and a reduction of adrenocorticotropin hormone as a result of massage therapy. Besides insula activation, CT fiber stimulation is also associated with the concomitant response of the nucleus tractus solitarius (NTS) directly linked to the paraventricular nucleus, responsible for the release of oxytocin (83). Baumgart et al. (2020) (46) explored CT fiber stimulation to reduce pain and comorbid depressive symptoms in somatoform disorder. Through the PRMT’s application, the authors obtained significant and continuous improvement in the depressive symptomatology in subjects with somatoform disorder, as well as a long-term effect on pain reduction (46). In addition, according to their projections to the amygdala, hippocampus, and cerebral cortex (84), oxytocin neurons may act through the neuromodulation of socio-emotional factors (stress and anxiety), which are known to influence pain perception (85).

## 5 Limitations

The present review highlights several limitations that should be considered to improve future research on affective touch in alleviating psychological distress. The first concern regards the limited number of studies available and the small sample sizes, which impact the reproducibility of findings and their applicability across different clinical populations. To further analyze the efficacy of affective touch interventions, it should be mandatory to expand the research with larger-scale studies. Another significant limitation is the heterogeneity of the psychological disorders that are characterized by distinct neurobiological and psychological mechanisms. Psychopathological differences and factors such as age, gender, cultural background, medication use, and comorbid conditions may strongly impact their response to affective touch and introduce potential confounding effects that can obscure the true impact of CT fiber stimulation. Moreover, the use of different protocols of stimulation complicates the comparability of findings and the identification of a comprehensive therapeutic approach even more. Moreover, this complicates the isolation of the specific effects of CT fiber stimulation. A key limitation of this study is the variability in methodological rigor among the included articles, as highlighted by the risk of bias assessment. While some studies demonstrated a low to moderate risk of bias, others presented significant methodological weaknesses, such as the absence of a control group, lack of randomization, and reliance on single-case designs. These limitations hinder the generalizability of the findings and emphasize the need for future research employing more robust study designs. The use of a more systematic approach characterized by standardized intervention guidelines (ensuring consistency in session duration, frequency, and intensity) could enhance the evaluation of the potential beneficial effects of affective touch in psychological disorders. The majority of existing studies focus on the short-term effects of affective touch, leaving its long-term potential effects unexplored. It could be useful to improve the research on this topic to build longitudinal studies with extended follow-up periods to evaluate if the observed beneficial effects persists over time. Despite the growing interest in the potential benefits of affective touch, the knowledge on the interactions of CT fiber stimulation with brain networks remains incomplete. While existing lines of evidence support the involvement of key regions such as the insula, amygdala, and reward circuitry, the precise pathways and the mechanisms through which affective touch exerts its effects are still not fully described yet. In addition, genetic and epigenetic influences on touch perception and response could offer insights into individual differences in affective touch. Furthermore, subjective effects associated with affective touch in emotional and cognitive dimensions when considering its potential therapeutic applications. By addressing these limitations, the field could progress, leading to a more comprehensive understanding of affective touch as a therapeutic tool. This will help to establish new clinical protocols of affective touch and expand its potential as a non-invasive intervention for individuals experiencing psychological distress.

## 6 Conclusion

This review explored the role of affective touch in psychological disorders, focusing on the potential modulation of CT fiber-mediated somatosensory processing. The evidence suggests that individuals diagnosed with psychiatric conditions tend to exhibit altered sensory perception, often reporting reduced perceived pleasantness of touch compared to healthy controls. These differences are attributed to disruptions in interoceptive processing and limbic system functioning.

In light of these findings, the review evaluated existing psychological interventions leveraging CT fiber stimulation. Despite methodological heterogeneity across the reviewed studies, a consensus emerged on the beneficial effects of affective touch therapies in reducing symptom severity and enhancing interoception in a wide range of psychological conditions.

Given the safety of these interventions and the current paucity of research, further studies are needed to explore their neuromodulatory effects and therapeutic potential, particularly in psychosis. Individuals with psychotic disorders often exhibit structural and functional alterations in the insular cortex, impairments in self–other differentiation, diminished interoceptive accuracy, attachment disturbances, and dysregulated stress responses. Affective touch may offer therapeutic benefits by modulating these key processes, warranting further investigation.

## Data Availability

The original contributions presented in the study are included in the article/supplementary material. Further inquiries can be directed to the corresponding author.
